# Convolutional neural network-based segmentation can help in assessing the substantia nigra in neuromelanin MRI

**DOI:** 10.1007/s00234-019-02279-w

**Published:** 2019-08-10

**Authors:** Alice Le Berre, Koji Kamagata, Yujiro Otsuka, Christina Andica, Taku Hatano, Laetitia Saccenti, Takashi Ogawa, Haruka Takeshige-Amano, Akihiko Wada, Michimasa Suzuki, Akifumi Hagiwara, Ryusuke Irie, Masaaki Hori, Genko Oyama, Yashushi Shimo, Atsushi Umemura, Nobutaka Hattori, Shigeki Aoki

**Affiliations:** 1 Department of Radiology, Juntendo University Graduate School of Medicine, 2-1-1, Hongo, Bunkyo-ku, Tokyo, 113-8421 Japan; 2 Department of Radiology, Université Paris Descartes, 12 rue de l’Ecole de Medecine, 75006 Paris, France; 3 Milliman Inc., Tokyo, Japan; 4 Department of Neurology, Juntendo University School of Medicine, Tokyo, Japan; 5 Department of Neurosurgery, Juntendo University School of Medicine, Tokyo, Japan

**Keywords:** Parkinson disease, Magnetic resonance imaging, Neural networks (computer), Artificial intelligence

## Abstract

**Purpose:**

This study aimed to evaluate the accuracy and diagnostic test performance of the U-net-based segmentation method in neuromelanin magnetic resonance imaging (NM-MRI) compared to the established manual segmentation method for Parkinson’s disease (PD) diagnosis.

**Methods:**

NM-MRI datasets from two different 3T-scanners were used: a “principal dataset” with 122 participants and an “external validation dataset” with 24 participants, including 62 and 12 PD patients, respectively. Two radiologists performed SNpc manual segmentation. Inter-reader precision was determined using Dice coefficients. The U-net was trained with manual segmentation as ground truth and Dice coefficients used to measure accuracy. Training and validation steps were performed on the principal dataset using a 4-fold cross-validation method. We tested the U-net on the external validation dataset. SNpc hyperintense areas were estimated from U-net and manual segmentation masks, replicating a previously validated thresholding method, and their diagnostic test performances for PD determined.

**Results:**

For SNpc segmentation, U-net accuracy was comparable to inter-reader precision in the principal dataset (Dice coefficient: U-net, 0.83 ± 0.04; inter-reader, 0.83 ± 0.04), but lower in external validation dataset (Dice coefficient: U-net, 079 ± 0.04; inter-reader, 0.85 ± 0.03). Diagnostic test performances for PD were comparable between U-net and manual segmentation methods in both principal (area under the receiver operating characteristic curve: U-net, 0.950; manual, 0.948) and external (U-net, 0.944; manual, 0.931) datasets.

**Conclusion:**

U-net segmentation provided relatively high accuracy in the evaluation of the SNpc in NM-MRI and yielded diagnostic performance comparable to that of the established manual method.

**Electronic supplementary material:**

The online version of this article (10.1007/s00234-019-02279-w) contains supplementary material, which is available to authorized users.

## Introduction

Parkinson’s disease (PD) is the second most common progressive neurodegenerative disease and affects 8.5 million individuals worldwide as of 2017 [1]. It is characterized by a progressive loss of dopaminergic neurons within the substantia nigra pars compacta (SNpc), considered to cause PD’s classical motor symptoms [2]. Currently, PD diagnosis relies on the clinical features acquired from patient history and neurological examination; accurate diagnosis is difficult in early stages, with a misdiagnosis rate of approximately 25% [3]. Although 60–80% of the dopaminergic neurons of the SNpc are lost before any clinical symptoms appear [4], to date, conventional MRI has been unsuccessful in detecting pathological changes in the SNpc, compromising the effectiveness of prophylactic approaches and new therapies [5] which attempt to slow the neuronal loss. Therefore, objective PD biomarkers are urgently desired.

In routine clinical practice, the role of MRI in patients with Parkinson-like motor symptoms is today limited to ruling out atypical parkinsonisms [6]. Recently, among other promising approaches [7, 8] developed to detect neurodegeneration in the SNpc, neuromelanin-MRI (NM-MRI) was proposed to visualize neuromelanin, as its depigmentation is a key pathological feature of PD [9]. Iron–neuromelanin complexes stored inside healthy dopaminergic neurons have highly paramagnetic properties that increase the NM-MRI signal intensity through a combination of magnetization transfer and T1 effects [10]. After neuronal death, unbound neuromelanin and iron become extracellular [11], contributing to neurodegeneration by activating the microglia and proinflammatory factors [12]. In patients with PD, low levels of intracellular iron–neuromelanin complexes result in decreased NM-MRI signal intensity. Several authors showed that quantifying the SNpc signal loss in NM-MRI can yield high diagnostic accuracy for distinguishing PD patients from controls [13–15], even at an early stage [16]. Furthermore, some studies reported a correlation with the severity of the disease [17, 18] and L-dopa induced motor complications [19].

To that purpose, various segmentation techniques have been proposed for assessing the hyperintense area of the SNpc: simple manual delineation [14], SNpc hyperintense area (or volume) estimation using a signal intensity-threshold derived from the manually segmented background midbrain [15, 18, 19], and the semiautomated region growing technique [20]. The only automated process described to date is the atlas-based method [13], which involves aligning new images to a set of manually labeled examples. However, this method may not be able to capture the full anatomical variability of the target subjects due to the use of a fixed set of atlases, affecting its accuracy [21], and is known to be computationally intensive.

In this study, we used as reference a threshold signal intensity method using manual segmentation (MS) first described by Schwarz et al. [15], as it is the only method demonstrating a stage-dependant SNpc signal loss in PD, unlike the atlas-based experiment. This method attempts to count the SNpc hyperintense pixels above a determined threshold based on the background signal of the midbrain. Several steps, including manually delineating the SNpc and midbrain, determining the threshold, and calculating the resulting hyperintense areas, are required. Despite attractive diagnostic performances, the clinical applicability of this method is impeded by these time-consuming steps, first and foremost MS; in this regard, automatized segmentation would be a significant improvement.

In this context, deep learning segmentation appears as an appealing option. It uses neural networks trained to perform tasks using examples and to grasp intricate structures in datasets [22]. Specifically, convolutional neural networks (CNNs) have significantly advanced computerized image recognition performance. They have successfully been applied to the neuroradiology field to segment various structures such as brain tumors [23], white matter hyperintensities [24], or organs-at-risks prior to radiation therapy [25]. Among CNNs, the U-net [26] is the most commonly used model in biomedical image segmentation.

We hypothesized that a U-net architecture CNN could replace manual segmentation of NM-MR images as the initial step of a previously described method aiming to assess SNpc signal intensity and achieve equivalent diagnostic accuracy for PD diagnosis. Therefore, we evaluated (1) the segmentation accuracy and (2) the diagnostic test performance of the U-net segmentation-based method compared to the established MS method.

## Methods

### Study design and participants

This retrospective case-control study used two NM-MRI datasets. A principal dataset from 60 patients with PD and 62 age- and gender-matched healthy controls (HC) was obtained by a 3T scanner (MAGNETOM Prisma, Siemens Healthcare) from October 2017 to July 2018 and was used to train and validate the U-net model. An external validation dataset, including 12 patients with PD and 12 HC, was obtained using a different 3T scanner (Achieva, Philips Medical Systems) from April 2014 to April 2015 and used to test the U-net. All patients were from the Neurology Department of Juntendo University Hospital and satisfied the Movement Disorder Society diagnostic criteria for clinically established PD [27]. These patients responded to antiparkinsonian therapy and remained free of atypical parkinsonism for 18 months or longer after being scanned. The HC group had no history of neurologic or psychiatric disorders. All the participants provided informed consent before examination and the Ethics Committee of the Juntendo University School of Medicine approved the study.

Using two 3T MR scanners, we obtained modified NM-sensitive T1-weighted fast-spin echo sequences with additional spectral presaturation inversion-recovery pulses, similar to that proposed by Schwarz et al. [15]. General scan parameters for the principal data set were as follows: 600/12 ms repetition time/echo time; echo train length of 14; 2.5 mm slice thickness; 0.5 mm slice gap; 3.0 mm spacing between slices; 512 × 359 acquisition matrix; 220 × 220 mm field of view (0.43 × 0.43 mm pixel size); 175 Hz/pixel bandwidth, three-averages; 7:15 min of total scan time, whereas those for the external validation dataset were as follows: 688/15 ms repetition time/echo time; echo train length of 14; 3 mm slice thickness; 1.0 mm slice gap; 4.0 mm spacing between slices; 0.43 × 0.43 mm pixel size; four-averages; 7:46 min of total scan time. In both cases, all the oblique-axial slices ranged from the splenium of the corpus callosum to the inferior border of the pons and were parallel to the line connecting the splenium to the genu of the corpus callosum and perpendicular to the fourth ventricle floor.

### Thresholding method based on manual segmentation

We used a similar, slightly modified version of the method reported by Schwarz et al. [15] to measure the hyperintense area in the SNpc. Image analyses were performed by two radiologists blinded to clinical information [reader 1 (KK) and reader 2 (AL)] on an offline Windows computer using the MRIcron software v2010. In this study, we used the masks delineated by the most experienced radiologist (reader 1) as criterion standard for SNpc and midbrain segmentation.

**Fig. 1:**
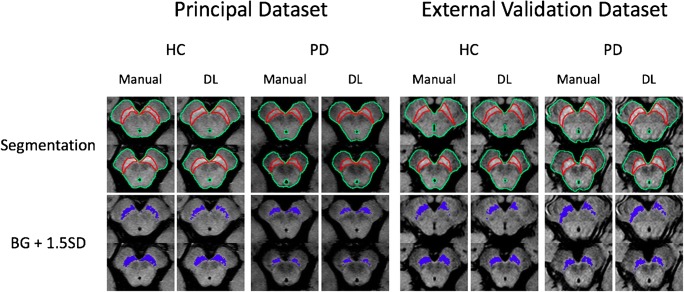
Manually labeled neuromelanin-sensitive MR images of midbrain and masks of hyperintense area within the SNpc obtained using the mean background signal intensity + 1.5 SD as a threshold*.* BG, background; DL, deep learning; HC, healthy control; PD, Parkinson’s disease

First, masks were generated by manually delineating the SNpc and midbrain in two consecutive axial slices that included the midbrain (Fig. 1). Then, we measured the average background signal and standard derivation (SD) for each patient, where the background was defined as the midbrain subtracted by the SNpc. The hyperintense areas of the SNpc were calculated by multiplying the number of pixels within the SNpc masks exhibiting signals above a chosen threshold by the image resolution. The optimal threshold was determined in the principal dataset by performing ROC analyses for the SNpc hyperintense areas using several thresholding values with intervals of the same order of magnitude as previous authors [18, 19]: MSI + 1, 1.5, or 2 SD. The highest diagnostic accuracy was yielded using the MSI + 1.5 SD threshold. We employed the same + 1.5 SD threshold in the external validation dataset, on the principle of externalizing both the segmentation and thresholding processes as a whole. This manual process took approximately 5 to 10 min for each subject.

### U-net architecture CNN-based segmentation

Deep learning segmentation was performed using a U-net architecture CNN [26] according to the maximum probability of candidate classes of each voxel. First, we augmented the principal dataset by changing the signal intensity, rotation degree, and scale of the original images to obtain a model that is robust against deviations, without prior signal intensity normalization. For signal intensity deviation, we converted the original signal intensity as

$$ A\ \left(\mathrm{orig}+B\right), $$

where *A* = 0.7/1.0/1.3, *B* = − 100/0/100, and orig denotes the signal intensity of the original image.

For rotational deviation, we processed the original image by rotating it by − 30°/0°/+ 30°. Finally, for scale deviation, we resized the original images to 90%/100%/110%. We used these augmented datasets as the final training data. Two U-nets were employed to perform two successive segmentation tasks, where one task was trained to segment the midbrain and the other was trained to segment the SNpc and background from the output of the former (i.e., midbrain = SNpc + background). The two U-nets had the same architecture, which differed slightly from the original U-net architecture [26]. Here, max pooling operations were replaced by convolution with a stride of 2. Both the U-nets were trained simultaneously to improve the second U-net’s robustness to noise, as output images from the first U-net exhibited significant noise; however, the loss functions of these U-nets were disconnected, ensuring that the backpropagation of the loss function of one U-net did not affect the other. In one iteration, a 256 × 256-pixel image was randomly cropped from the original data (512 × 512 pixels) with a batch size of 2, and mean cross entropy was computed for the loss function. The Adam [28] gradient descent algorithm was used to optimize the model. Here, the Adam update rule was applied with *α* = 0.0001, *β*1 = 0.5, and *β*2 = 0.999. Adam optimization was performed for 10 epochs. Training of the U-net was performed according to a 4-fold cross-validation based on four balanced groups mixing HC and PD patients with a 1:1 ratio. Validation and training Dice coefficients were similar in all folds; therefore, the model trained on the first fold was arbitrarily chosen to perform U-net segmentation (US). We did not employ early stopping or hyper parameter search.

As an inference phase, the trained U-net was applied to the external validation dataset to segment the unlabelled Achieva MR images. NM-rich areas of the SNpc were calculated using the optimal threshold obtained with the principal dataset (signal intensity of the midbrain +1.5SD).

A computer with 64 GB of CPU memory, a Xeon E5-2670 v3 CPU (Intel, Santa Clara, CA), and a TITAN Xp graphics processing unit (NVIDIA, Santa Clara, CA) was used to perform the model training. Python 3.6 and the DL framework of Chainer 3.2.0 (http://chainer.org/) was used to code the computer program. Each fold took 150 min to process. The time to predict (U-net segmentation and hyperintense voxels count) for each patient was less than 0.5 s.

### Statistical analyses

Statistical analyses were performed using the XLSTAT v2018.7 software. Age and gender distributions were compared between patients with PD and HC using Student’s *t* test and chi-squared test. The relative variation of the background signal was calculated by dividing the mean background signal with the standard deviation. The mean relative variations in the HC and PD groups were compared using the Student’s *t* test to assess the image quality. To evaluate the segmentation accuracy of the U-net, we used the Dice similarity coefficient (DSC) defined as$\mathrm{Dice}\ \left(\mathrm{MS},\mathrm{US}\right)=\frac{2\mid \mathrm{MS}\cap \mathrm{US}\mid }{\left|\mathrm{MS}\right|+\left|\mathrm{US}\right|}$, where MS denotes manual segmentation and US denotes U-net segmentation. DSC values range from 0 to 1, where 0 and 1 indicate no and perfect overlapping, respectively. We compared the segmentation outputs of the U-net to the masks delineated manually by reader 1 (KK), considered the criterion standard. We also compared the two readers’ segmentation masks. The inter-reader precision and segmentation accuracy of the U-net was rated as follows: 0.0–0.39, “low”; 0.40–0.79, “moderate”; and 0.80–1.0, “high.” In terms of DSC, using Mann–Whitney *U* test and Student’s *t* tests with the principal and external validation datasets, respectively, we compared the U-net accuracy or the inter-reader precision for SNpc or midbrain segmentation between the HC and PD groups. In addition, we compared the U-net accuracy to inter-reader precision for SNpc segmentation in both the HC and PD groups. The Student’s *t* test was used to compare the NM-rich areas of the SNpc between the PD and HC groups. The relations between the hyperintense areas and disease duration or UPDRS-III scores were determined using the Spearman’s rank correlation test. The strength of the correlation was determined using the following criteria for correlation coefficient r: 0.00–0.19, “very weak;” 0.20–0.39, “weak;” 0.40–0.59, “moderate;” 0.60–0.79, “strong;” 0.80–1.0, “very strong.” Finally, to evaluate the diagnostic performance of the thresholding method as a diagnostic test for PD using either manual or U-net segmentation, receiver operating characteristic (ROC) analyses of hyperintense SNpc areas were performed and areas under the curve (AUC) calculated. ROC curves form the same datasets were compared using the Delong method [29].

## Results

### Participants and image quality

There was no significant difference in age or gender between the HC and patients with PD in the two datasets (Table 1). Further, there was no significant difference in the relative variations of the background signals between the patients with PD and HC in both datasets for either segmentation method (all *p* > 0.05, see supplemental data online), indicating that the image qualities were similar in the PD and HC groups.

**Table 1:** Clinical characteristics of healthy controls and PD patients in the principal and external validation datasets

	Principal dataset			External validation dataset		
Variable	HC, *n* = 60	PD, *n* = 62	*p* value	HC, *n* = 12	PD, *n* = 12	*p* value
Gender (m/f)	35; 25	28; 34	0.146	12; 0	12; 0	N.A.
Age, year	70.82 ± 3.68	70.24 ± 6.45	0.549	62 ± 14.33	62.5 ± 9.69	0.947
Disease duration, year	N.A.	9.98 ± 6.22		N.A.	15.75 ± 13.33	
UPDRS-III score	N.A.	22.47 ± 15.67		N.A.	20.08 ± 9.88	
Hoehn and Yahr stage	N.A.	2.73 ± 0.87		N.A.	2.5 ± 0.52	

Data are presented as mean ± standard deviation unless otherwise noted

*HC*, healthy controls; *PD*, Parkinson’s disease; *UPDRS-III*, part III of the Unified Parkinson’s Disease Rating Scale

### Evaluation of the U-net

#### Principal dataset

**Fig. 2:**
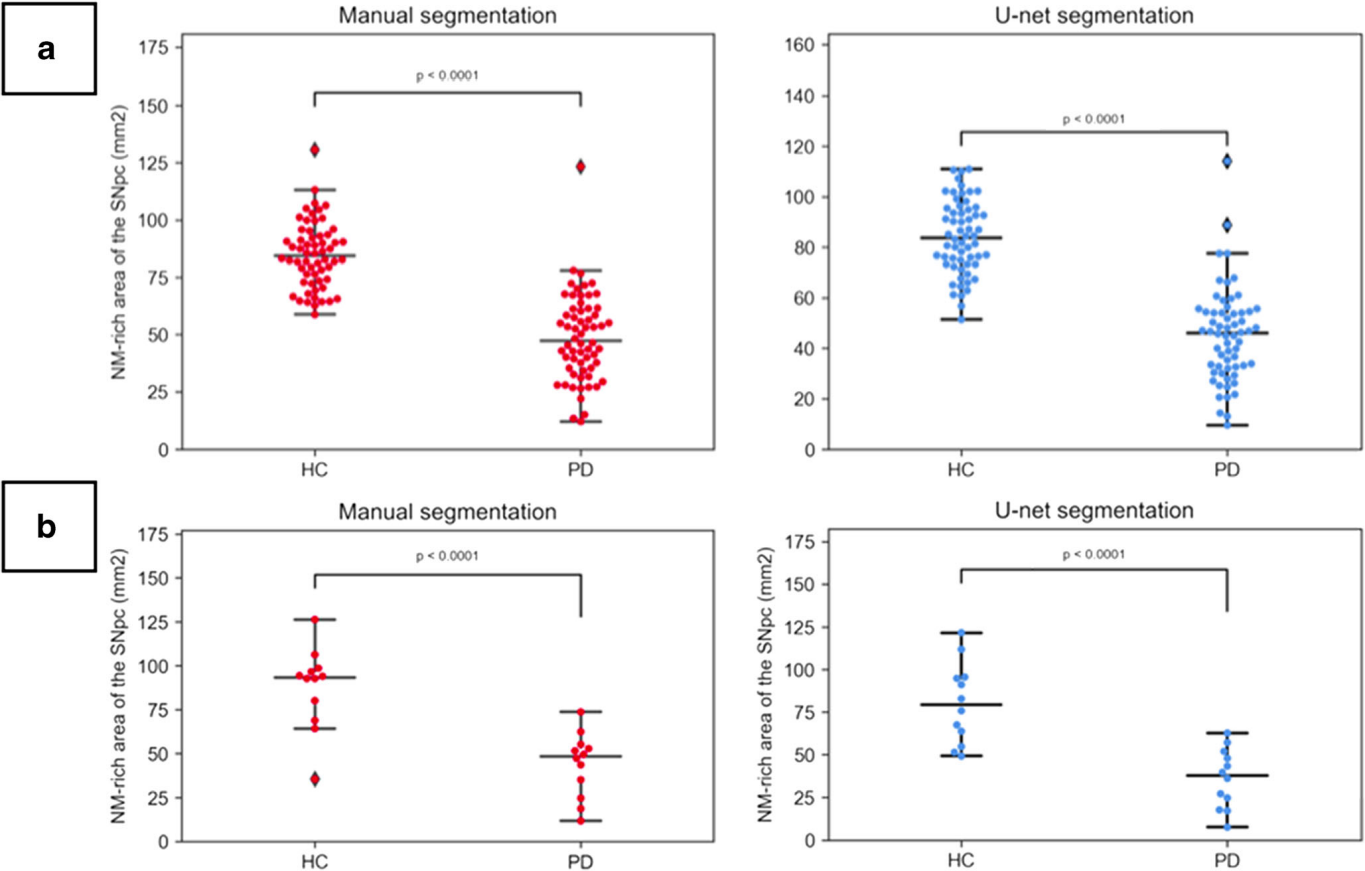
Neuromelanin-rich areas obtained with manual (reader 1) and U-net segmentation methods on neuromelanin-sensitive MR images of the principal (**a**) and external validation (**b**) datasets

**Fig. 3:**
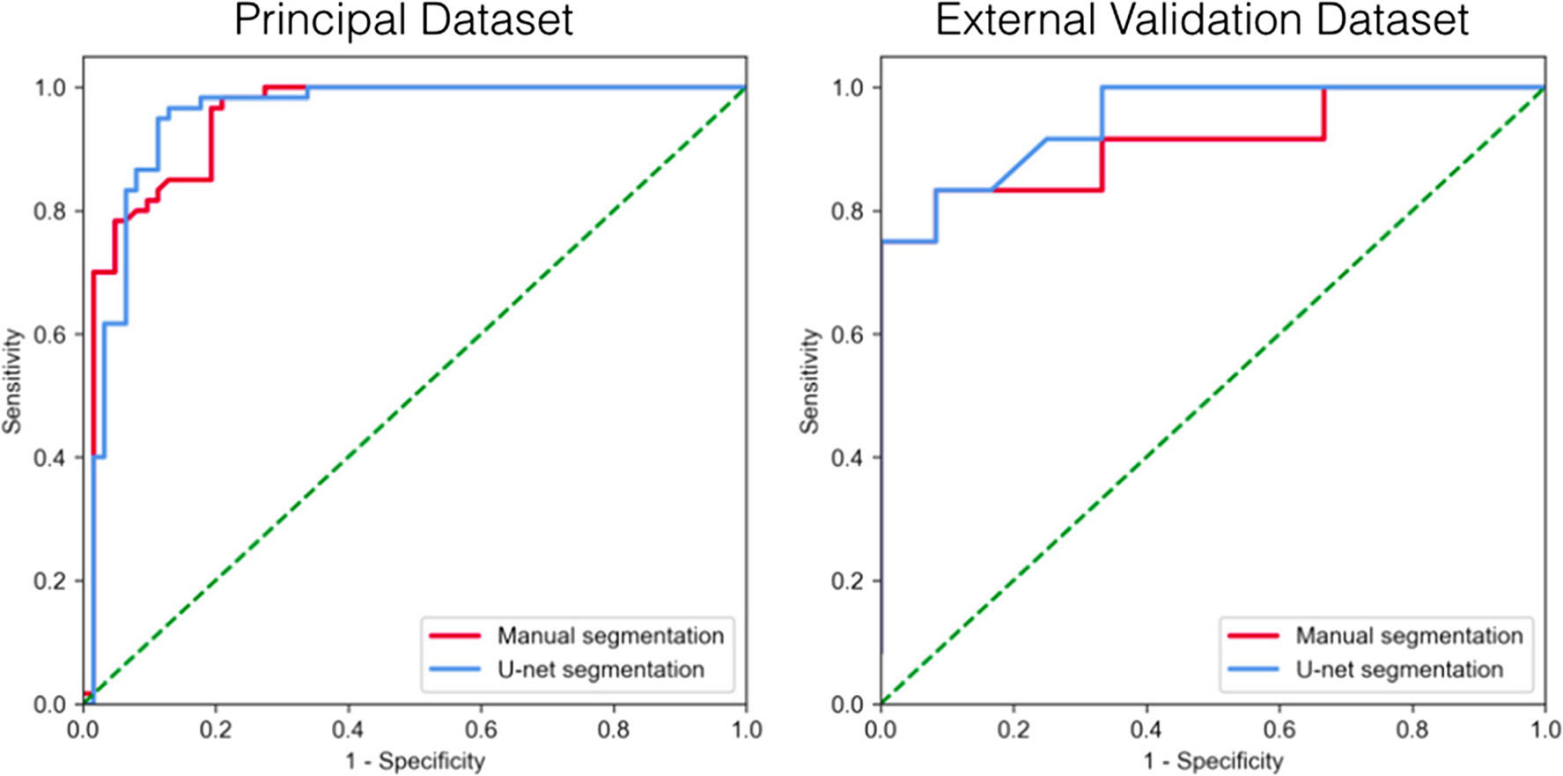
ROC curves of NM-rich areas of the SNpc obtained by manual and U-net segmentations for differentiating patients with PD and healthy subjects in both datasets

Table 2 shows the inter-reader precision and US accuracy in terms of the DSC obtained for the SNpc and midbrain. The US accuracy of SNpc and midbrain (MB) was as high as the inter-reader precision, with similar DSCs for each subgroup and structure. The DSCs were consistently lower for the SNpc than for the midbrain (DSCs for all subjects: midbrain, 0.97 ± 0.01; SNpc, 0.83 ± 0.04). Further, regarding the SNpc, the DSCs were lower for patients with PD than HC. The calculated hyperintense areas within the SNpc were significantly lower in the patients with PD than in the HC [MS: PD, 48.6 ± 19.1 mm^3^ (mean ± SD); HC, 84.9 ± 14.4 mm^3^; US: PD, 45 ± 18.5 mm^3^; HC, 83.9 ± 14.5 mm^3^; all *p* < 0.0001 using Student’s *t* test] (Fig. 2a). Analysis of the correlation between the hyperintense area and PD duration demonstrated a significantly weak negative correlation with both methods (MS: *r* = − 0.32, *p* = 0.013; US: *r* = − 0.33, *p* = 0.008) but no significant correlation with the UPDRS-III score (MS: *r* = − 0.22, *p* = 0.079; US: *r* = − 0.20, *p* = 0.110). Using US instead of MS for the threshold signal intensity method did not affect the diagnostic test performance of NM-MRI. The AUCs for the hyperintense SNpc area were comparable using either the US or MS methods with a slight comparative advantage for US (AUCs, 0.950 and 0.948, respectively, *p* < 0.05, with optimal cut-off values of 61.2 and 64.2 mm^2^, Fig. 3).

**Table 2:** Inter-reader precision and U-net segmentation accuracy shown as mean DSC obtained for the SNpc and midbrain in healthy controls and PD patients of both datasets

Dataset	Structure	Mean DSC Reader 1 versus reader 2				Mean DSC U-net versus reader 1			
		All	HC	PD	*p* value*	All	HC	PD	*p* value*
Principal	MB SNpc	0.97 ± 0.01 0.83 ± 0.04	0.97 ± 0.01 0.85 ± 0.04	0.97 ± 0.01 0.81 ± 0.04	0.974 < 0.001	0.97 ± 0.01 0.83 ± 0.04	0.97 ± 0.01 0.84 ± 0.03	0.97 ± 0.02 0.82 ± 0.04	0.868 0.001
External validation	MB SNpc	0.96 ± 0.02 0.85 ± 0.03	0.95 ± 0.02 0.86 ± 0.02	0.96 ± 0.02 0.83 ± 0.03	0.563 0.036	0.95 ± 0.01 0.79 ± 0.04	0.96 ± 0.01 0.80 ± 0.05	0.95 ± 0.02 0.77 ± 0.03	0.532 0.101

Data are presented as mean ± standard deviation unless otherwise noted

*DSC*, Dice similarity coefficient; *HC*, healthy controls; *MB*, midbrain; *PD*, Parkinson’s disease; *SNpc*, substantia nigra pars compacta

*Between HC and PD patients

#### External validation dataset

Visual assessment of the output segmentation maps did not reveal any large segmentation errors. The US accuracy for SNpc was high for HC and moderate for patients with PD but was significantly lesser than the inter-reader precision in both cases (mean DSCs: patients with PD, 0.77 versus 0.83; HC, 0.80 versus 0.86, all *p* < 0.0001). As with the principal dataset, the SNpc segmentation was less accurate than the midbrain segmentation in each case (Table 2). Hyperintense areas were significantly lower in the patients with PD than HC, and the differences between these groups were similar with both methods [MS: PD, 43.9 ± 18.2 mm^3^; HC, 87.6 ± 23.1 mm^3^; US: PD, 36.1 ± 17.4 mm^3^; HC, 80 ± 23.7 mm^3^; all *p* < 0.0001 using Student’s *t* test] (Fig. 2b). No significant correlation was found between the hyperintense area and disease duration (MS: *r* = − 0.24, *p* = 0.449; US: *r* = − 0.20, *p* = 0.527) with either method. However, there was a strong significant correlation with the UPDRS-III score (MS: *r* = − 0.65, *p* = 0.027; US: *r* = − 0.60, *p* = 0.043). Here too, replacing MS by US did not seem to affect the overall diagnostic test performance of NM-MRI. The AUCs for the hyperintense SNpc area were respectively 0.944 and 0.931 when US or MS were employed (*p* < 0.05), with optimal cut-off values of 54.7 and 64.3 mm^2^ (Fig. 3).

## Discussion

Here, we developed a U-net model to segment the SNpc and midbrain in NM-MRI and showed that our model could achieve equivalent diagnostic performance to that of manual segmentation using a validated thresholding method for the hyperintense area of the SNpc, despite a moderate segmentation accuracy of the SNpc by our model.

U-net segmentation of the midbrain was highly accurate in both datasets; however, the U-net could not achieve a segmentation of the SNpc in the same range as the inter-reader precision in the external validation dataset. The lower accuracy of the US for SNpc in the external dataset implies that different imaging parameters and signal intensity variations challenge the U-net inference capabilities. Also, applying the optimal threshold for the principal dataset to the external dataset could have affected the diagnostic test accuracy, because the threshold should be adapted to the neuromelanin-sensitivity level of the pulse sequence. To address this specific issue, Schwarz et al. [18] proposed a normalization procedure based on the theoretical volume of the SNpc hyperintense area in healthy controls. Because we wanted to test independently the accuracy of the U-net in the external dataset, we did not try to normalize the signal intensity level.

Another finding is that the accuracy of the SNpc segmentation was consistently lower than that of the midbrain, denoting the difficulty in determining the boundary of the SNpc regardless of the segmentation method. Unlike the boundaries between the midbrain and surrounding cisterns, the boundaries between the SNpc and the background are difficult to delineate precisely because hyperintense pixels depict only neuromelanin content and not the entire SNpc. The relative subjectivity inherent in the manual segmentation of the SNpc seems to have affected both manual and U-net segmentation accuracies. Further, the DSCs were lower in the patient group compared to the healthy group probably because reduced-hyperintense areas result in an even more challenging segmentation task.

Despite the relative lack of precision of the SNpc segmentation in the external dataset, the calculated hyperintense areas were significantly reduced in patients with PD compared to HC in both datasets, consistent with the results of previous studies [13, 15, 19, 20]. The diagnostic test accuracy for PD of the thresholding method was not affected: AUC were similar using U-net or manual segmentation in both datasets, with a slight comparative advantage for the U-net method, and as high to that of the previously described manual techniques, where it ranged from 0.82 to 0.93 [13, 15, 20]. These results suggest that an extremely precise segmentation of the SNpc is not required to provide useful size estimates of the hyperintense area. Our U-net model is sufficient to obtain a satisfying diagnostic accuracy.

The hyperintense areas were correlated to motor severity (reflected by UPDRS-III scores) in the external validation dataset but not in the larger principal dataset. We do not have a clear explanation for this finding, as disease severity was similar between the two groups. Due to its small size (12 PD patients), the correlation analyses performed on the external validation dataset should be viewed cautiously. As previous studies on smaller samples also found weak [18] or no correlation [13, 17] with UPDRS-III scores, the utility of NM-MRI as a monitoring tool for patients with PD could not be proved.

This study had several limitations. First, the sample size was relatively small for a case-control study, particularly of the external validation dataset. Second, PD diagnosis in this study was not histopathologically confirmed; thus, misdiagnosis could be possible. Third, as pathological examination could not be used as a criterion, the U-net model was trained using manually obtained masks of the SNpc and midbrain from NM-MRI as input. MS relies on recognition of the hyperintense area and the anatomical knowledge of the radiologist and is therefore subject to subjectivity bias. Hyperintense areas could be underestimated in patients with PD, amplifying the difference between the patients with PD and HC. Thus, additional sequences providing clearer SNpc images, such as proton density-weighted images, could be beneficial for creating more accurate SNpc masks for application to NM-MR images. Fourth, both the methods relied on a threshold to define the hyperintense area. A drawback of this approach is the loss of information, such as the magnitude of the signal intensity above the threshold or its spatial distribution [30]. Several studies have found sub-regional patterns of neuromelanin loss within the SNpc using manually placed regions of interest [16, 18] or voxel-wise analysis [30], with differences between HC and patients with PD preferentially involving the posterior and lateral parts of the SNpc. Studying the whole SNpc could have contributed to the lack of correlation with the clinical status in our study, which remains an important focus for further improvement of NM-MRI. Additional studies focusing on this region of the SNpc could help achieve this goal. Finally, mean disease duration was longer in the external validation dataset and it may have influenced positively the diagnostic accuracy of the method in the external dataset. Additionally, the mean age differed between the datasets; thus, the potential influence from these factors cannot be ignored because the midbrain is subject to age-related changes [31]. However, despite these limitations, because the U-net saves times and does not affect the diagnostic accuracy of the thresholding method, it may be useful to promote the clinical application of NM-MRI for PD diagnosis.

In conclusion, U-net segmentation provided relatively high accuracy in the evaluation of the SNpc in NM-MRI and yielded diagnostic performance comparable to that of the established manual method, but its segmentation accuracy should be further improved to be able to fully replace manual segmentation.

## Electronic supplementary material

ESM 1 (DOCX 16 kb)
