# Supplementary material for: Convolutional neural network-based segmentation can help in assessing the substantia nigra in neuromelanin MRI
Source: Neuroradiology. 2019 Aug 10;61(12):1387–95. doi: 10.1007/s00234-019-02279-w (PMC6848644; doi:10.1007/s00234-019-02279-w)
Supplement: Supplementary file 1 — (DOCX 16 kb) [file 234_2019_2279_MOESM1_ESM.docx]

**Supplemental online data**:

Relative variation of the background signal of the midbrain obtained in PD patients and HC after manual or U-net segmentation in principal or external validation datasets

| Dataset | Segmentation method | Relative variation of the background signal | | P value | |
| --- | --- | --- | --- | --- | --- |
|  |  | HC | PD patients | |  |
| **Principal** | Manual  U-net | n = 60  14.00 ± 2.09  13.66 ± 1.07 | n = 62  14.41 ± 2.11  13.27 ± 1.31 | | 0.290  0.074 |
| **External Validation** | Manual  U-net | n = 12  15.37 ± 2.43  15.66 ± 1.67 | n = 12  16.97 ± 2.69  15.94 ± 1.81 | | 0.143  0.703 |

*HC, healthy controls; PD, Parkinson’s disease*

Data are presented as mean ± standard deviation unless otherwise noted.
